# The lncRNA–DNA Methylation Axis in Hepatocellular Carcinoma: Mechanisms, Epigenetic Plasticity, and Biological Implications

**DOI:** 10.3390/biology15060458

**Published:** 2026-03-11

**Authors:** Lingke Meng, Lingzhu Cheng, Yuanyuan Li, Yushan Guo, Na Li

**Affiliations:** Department of Pathophysiology, College of Basic Medical Sciences, Jilin University, Changchun 130021, China; menglk25@mails.jlu.edu.cn (L.M.); chenglz24@mails.jlu.edu.cn (L.C.); yuanyuanl25@mails.jlu.edu.cn (Y.L.); guoys23@mails.jlu.edu.cn (Y.G.)

**Keywords:** hepatocellular carcinoma, long non-coding RNA, DNA methylation, epigenetics, epigenetic plasticity, DNMT, TET, therapeutic resistance, EMT

## Abstract

DNA methylation is a fundamental epigenetic regulator in hepatocellular carcinoma (HCC). However, a key paradox remains: how do ubiquitously expressed enzymes like DNMTs and TETs achieve locus-specific regulation without intrinsic sequence specificity? This review aims to elucidate the “lncRNA–DNA methylation axis,” examining how long non-coding RNAs (lncRNAs) confer specificity and plasticity to methylation machinery. We synthesized current literature focusing on the structural mechanisms (e.g., R-loops, DNA:RNA triplexes) by which lncRNAs interact with DNMTs and TETs. We further analyzed the bidirectional regulation between lncRNAs and methylation enzymes and their impact on HCC phenotypes. lncRNAs function as modular scaffolds and guides, directing methylation machinery to specific genomic loci. Rather than binary switches, they act as an “epigenetic rheostat,” fine-tuning methylation intensity to balance stability with plasticity. Crucially, a reciprocal feedback loop exists: aberrant DNA methylation suppresses tumor-suppressive lncRNAs, which in turn unleashes DNMT activity, locking cells into a malignant state. This axis drives proliferation, metastasis, metabolic reprogramming, and therapeutic resistance. The lncRNA–DNA methylation axis is a central determinant of epigenetic heterogeneity in HCC. Moving beyond descriptive cataloging to a mechanistic understanding of this network offers new perspectives for developing targeted epigenetic therapies and biomarkers.

## Simple Summary

Hepatocellular carcinoma (HCC) is a deadly liver cancer driven by complex molecular changes. While genetic mutations are well-studied, the role of “epigenetics”—chemical modifications that control gene activity without changing the DNA code—is equally critical. This review explores how a special class of RNA molecules, called “long non-coding RNAs” (lncRNAs), acts as a bridge to guide DNA methylation machinery to specific genes. Instead of functioning as simple on/off switches, these lncRNAs fine-tune gene expression like a dimmer switch, helping cancer cells survive, spread, and resist treatment. We also discuss how abnormal methylation can silence beneficial lncRNAs, creating a vicious cycle that promotes tumor growth. Understanding this two-way relationship offers new hope for developing better biomarkers and targeted therapies for liver cancer patients.

## 1. Introduction

Hepatocellular carcinoma (HCC) remains one of the leading causes of cancer-related mortality worldwide, largely due to its aggressive biological behavior, high recurrence rate, and limited therapeutic options for advanced disease [1]. Despite substantial progress in understanding the genetic landscape of HCC, genetic alterations alone fail to fully explain the pronounced phenotypic heterogeneity and adaptive capacity observed during tumor initiation, progression, and treatment resistance [2]. Increasing attention has therefore been directed toward epigenetic dysregulation—particularly aberrant DNA methylation—as a fundamental driver of hepatocarcinogenesis [3].

Among epigenetic mechanisms, DNA methylation represents a central regulatory layer governing gene expression, chromatin organization, and genome stability [4,5]. Aberrant DNA methylation patterns, characterized by global hypomethylation alongside locus-specific hypermethylation of tumor suppressor genes, are hallmarks of HCC [6]. Dysregulation of DNA methyltransferases (DNMTs) and ten–eleven translocation (TET) dioxygenases has been extensively documented, underscoring the importance of dynamic methylation–demethylation balance in liver cancer [7,8]. Notably, DNMTs and TET enzymes lack intrinsic DNA sequence specificity. A critical question, therefore, remains unresolved: how are these enzymatic activities precisely directed to selected genomic loci to establish cancer-specific methylation landscapes? This is particularly pertinent given emerging evidence that long non-coding RNAs play diverse regulatory roles in recruiting or modulating DNA methylation machinery across the genome.

Recent advances in single-cell and spatial epigenomic profiling have further revealed profound heterogeneity in DNA methylation states across tumor subpopulations, underscoring the need for regulatory mechanisms that enable both locus specificity and cellular plasticity [9,10]. Long non-coding RNAs (lncRNAs) have emerged as key regulators of gene expression and chromatin dynamics, exerting diverse biological functions without encoding proteins [11]. In HCC, numerous lncRNAs have been implicated in tumor growth, metastasis, metabolic reprogramming, and immune modulation [12,13]. Mechanistically, lncRNAs are particularly well suited to act as molecular scaffolds, guides, and spatial organizers within the nucleus, enabling them to interface with chromatin-modifying enzymes and nuclear architecture [14,15,16].

Despite increasing recognition of lncRNA–DNA methylation interactions in HCC, several fundamental questions remain unresolved. First, how lncRNAs confer locus specificity to DNMTs and TET enzymes in the absence of intrinsic DNA-binding capacity remains incompletely understood [17]. Emerging evidence suggests that structural interfaces, including R-loops and DNA:

RNA triplexes may serve as potential platforms facilitating the recruitment or modulation of DNA methylation machinery, although their functional relevance and context dependency in HCC require further clarification [18]. Second, whether lncRNA-mediated DNA methylation remodeling functions predominantly as stable, heritable drivers of tumor evolution or as dynamic, context-dependent modulators responsive to environmental, metabolic, or oncogenic cues remains an open question [19]. Third, it is unclear to what extent these interactions operate as coordinated, network-level regulatory programs rather than isolated lncRNA-centered events, particularly in shaping epigenetic plasticity and intratumoral heterogeneity in HCC [20]. Addressing these unresolved issues is essential for establishing a mechanistic framework that integrates lncRNA function with dynamic DNA methylation remodeling and for evaluating their implications in hepatocarcinogenesis, biomarker development, and therapeutic intervention.

## 2. lncRNA-Mediated Regulation of DNA Methylation in HCC

### 2.1. Recruitment of DNMTs by lncRNAs

A major mechanism involves lncRNAs functioning as molecular scaffolds that bridge *DNMTs* with chromatin-associated complexes [21]. *HOTAIR* represents a prototypical scaffold lncRNA in HCC, interacting with DNMT1 and DNMT3B to promote promoter hypermethylation and stable silencing of tumor suppressor genes [22,23] (Table 1). Similar scaffold-based interactions have been reported for *ANRIL* and related lncRNAs, primarily through stabilizing repressive chromatin complexes, which may facilitate DNMT-dependent DNA methylation [24].

In addition to scaffolding functions, some lncRNAs can act in a guide-like manner to direct DNA methyltransferases to particular genomic loci. For instance, *NEAT1* has been shown to bind DNMT1 and enhance its enrichment at promoters of key tumor suppressor genes such as *TP53*, *cGAS* and *STING*, leading to locus-specific DNA methylation. While *TUG1* and other lncRNAs are implicated in epigenetic regulation, their interaction with the DNA methylation machinery is generally considered indirect—they primarily scaffold intermediate chromatin-modifying complexes (such as PRC2) rather than physically anchoring *DNMTs* to DNA [25,26]. Beyond linear genome targeting, nuclear architecture-associated lncRNAs such as *MALAT1* modulate DNMT activity indirectly. Rather than directly binding methylation enzymes, *MALAT1* shapes subnuclear compartments (e.g., nuclear speckles) to alter the spatial distribution and local enrichment of these epigenetic regulators [27,28,29] (Table 1). Together, these mechanisms illustrate how lncRNAs enable precise and efficient DNMT-mediated methylation remodeling in HCC.

Despite the mechanistic elegance of the scaffold and guide models demonstrated by paradigmatic lncRNAs like *HOTAIR* and *NEAT1*, it is essential to contextualize their broad applicability in HCC. Transcriptomic and interactomic studies have increasingly revealed that lncRNA functions are exceptionally context-, locus-, and cell-state-dependent [30]. Crucially, large-scale RNA-protein interactome analyses suggest that among the thousands of dysregulated lncRNAs in cancer, only a marginal fraction possesses the specific secondary structural motifs required for high-affinity, direct physical engagement with the DNA methylation machinery [31]. Instead, a substantial proportion of methylation-associated lncRNAs in HCC operate through indirect mechanisms—such as scaffolding broad chromatin-modifying complexes (e.g., EZH2/PRC2), which subsequently recruit DNMTs to establish repressive chromatin states [32] (Table 1). Consequently, while prototypical lncRNAs provide valuable mechanistic insights, extrapolating their direct scaffolding or guiding capacities to the broader lncRNA transcriptome without rigorous structural and biochemical validation risks oversimplifying the highly orchestrated epigenetic networks in HCC.

### 2.2. lncRNA Regulation of DNA Demethylation Pathways

DNA methylation in HCC is dynamically counterbalanced by active demethylation mediated by ten–eleven translocation (TET) enzymes [33,34]. Emerging evidence indicates that lncRNAs participate in this process by modulating TET expression, protein stability, and chromatin association, thereby influencing the overall capacity for DNA demethylation [17,35].

In the context of HCC, the regulation of TET enzymes by lncRNAs frequently occurs at the post-transcriptional level or through metabolic interference, rather than via direct physical scaffolding. For example, several HCC-associated lncRNAs act as competing endogenous RNAs (ceRNAs) to sponge miRNAs that would otherwise degrade *TET1* or *TET2* transcripts, thereby indirectly restoring active DNA demethylation at tumor suppressor loci. Furthermore, lncRNAs such as *H19* have been reported to modulate methylation dynamics in HCC by interacting with S-adenosylhomocysteine hydrolase (SAHH), indirectly altering the biochemical environment required for TET and DNMT activities without acting as direct targeting guides. Beyond altering transcript abundance or metabolic states, the direct physical recruitment of TET enzymes to chromatin by lncRNAs represents a critically important mechanism for locus-specific demethylation. Importantly, while definitive structural evidence for direct lncRNA-TET scaffolding in HCC remains limited, elegant studies from other solid tumors provide a highly plausible mechanistic blueprint. For instance, in breast cancer, the lncRNA *TARID* has been experimentally proven to bind GADD45A, stabilizing R-loop structures to directly guide TET1 to the *TCF21* promoter, thus preventing *de novo* methylation and facilitating transcriptional activation [35,36] (Table 1). Similarly, emerging evidence in related gastrointestinal malignancies suggests that specific lncRNAs can physically anchor TET proteins to target genomic loci. We propose that analogous spatial guidance mechanisms likely operate during hepatocarcinogenesis, highlighting a crucial avenue for future spatial epigenomic research in HCC.

Importantly, lncRNAs often regulate both DNMT- and TET-mediated pathways, acting as dynamic modulators rather than unidirectional regulators [37]. This coordinated control enables reversible and context-dependent methylation changes, supporting epigenetic plasticity during HCC progression [38].

### 2.3. Molecular Basis of lncRNA-Mediated Locus Specificity

**Figure 1:**
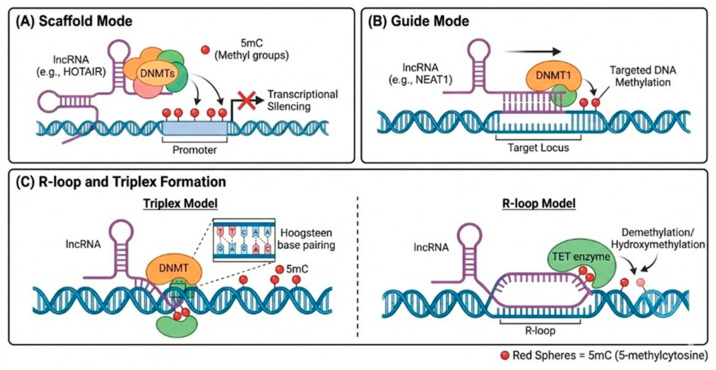
Canonical modes of lncRNA-mediated regulation of DNA methylation in HCC. (**A**) Scaffold model: lncRNAs (e.g., *HOTAIR*) act as adaptors bridging DNMTs with chromatin. (**B**) Guide model: lncRNAs recruit DNMTs to specific loci via DNA:RNA triplexes or R-loops. (**C**) Demethylation regulation: lncRNAs modulate TET enzyme stability or recruitment. Note: While direct scaffolding and guiding mechanisms have been experimentally validated for prototypical lncRNAs like *HOTAIR*, the extrapolation of these models as universal pathways across the transcriptome remains conceptual. Most lncRNA interactions with the methylation machinery are likely indirect and highly context-dependent.

Beyond acting as simple protein-tethers, lncRNAs employ sophisticated biochemical strategies to ensure locus-specific DNA methylation patterns. Emerging evidence suggests that certain lncRNAs, such as *GADD45A*-associated transcripts, facilitate TET1 recruitment by stabilizing R-loop structures at specific CpG island promoters, thereby preventing *de novo* methylation and maintaining a transcriptionally active state [36,39]. Furthermore, certain lncRNAs can engage in sequence-specific recognition of the DNA major groove, forming RNA:DNA triplexes via Hoogsteen or reverse-Hoogsteen base pairing. This mechanism provides a high-affinity physical anchor for *de novo* DNMTs at specific genomic loci, particularly those lacking canonical protein-binding motifs, thereby ensuring precise epigenetic silencing [40] (Figure 1). Importantly, while these sophisticated structural interfaces (e.g., RNA:DNA triplexes and R-loops) represent highly conserved fundamental mechanisms universally applicable across various cancer types, their ultimate oncogenic consequences are profoundly HCC-specific. The unique lncRNA interactome synthesized within the hepatic microenvironment ensures that these universal structural tools are exclusively deployed to methylate hepatocyte-specific tumor suppressors. In the context of HCC, effectively translating these theoretical structural models into validated epigenetic mechanisms requires rigorous experimental precision. Current computational predictions of lncRNA-mediated targeting must be substantiated by specialized genomic techniques, such as DRIP-seq (DNA:RNA immunoprecipitation sequencing) or its refined adaptations (e.g., DRIP-seq, R-ChIP, CUT&Tag) for mapping R-loops [41,42], and Triplex-seq for RNA:DNA triplexes [43]. However, it is crucial to recognize common experimental confounders; for example, predicted structural motifs do not inherently equate to functional DNMT or TET recruitment in vivo. To establish true causality rather than correlative chromatin occupancy, studies must employ functional perturbations. Demonstrating that the targeted application of RNase H (which specifically degrades the RNA strand in RNA:DNA hybrids) or the mutational disruption of triplex-forming nucleic acid motifs directly abolishes DNMT/TET recruitment and reverses downstream methylation at the predicted loci is an essential standard [44]. Such stringent validation workflows—from mapping structural interfaces to verifying physical interaction, localized recruitment, quantifiable methylation change, and ultimately the biological phenotype—are critically needed in HCC research to distinguish bona fide targeting guides from mere transcriptional byproducts [45].

### 2.4. Integrated Regulatory Model

Building on emerging evidence for structural RNA–DNA interfaces, including R-loops and DNA:RNA triplexes, lncRNA-mediated DNA methylation regulation in HCC is increasingly viewed not as a binary on–off switch, but as a multi-layered and context-dependent regulatory network. Through interactions with DNMTs and TET enzymes, lncRNAs have been implicated in multiple phases of DNA methylation dynamics, including the establishment, maintenance, and reversal of methylation marks, often in a context-dependent manner [17,19].

Within this framework, lncRNAs act as ‘epigenetic rheostats’ that quantitatively fine-tune methylation intensity and spatial distribution. Operationally, rather than functioning as strictly binary (0% or 100%) silencing switches, this rheostat behavior is defined by the capacity to establish and maintain graded, intermediate DNA methylation states (e.g., maintaining 30–40% versus 70–80% CpG methylation density at a specific promoter) or to drive measurable cell-to-cell epigenetic variance within a tumor population [20,46]. In HCC, testing and validating this rheostat model relies critically on emerging single-cell bisulfite sequencing (scBS-seq) and spatial methylomics. These technologies can reveal continuous spectra of epigenetic states that biologically enable highly reversible, partial phenotypic shifts—most notably, the hybrid epithelial–mesenchymal transition (EMT) states critical for HCC metastasis [47,48]. To definitively validate or falsify this model, future experimental designs must employ dose-dependent perturbations (e.g., using tunable CRISPR interference to gradually titrate lncRNA expression) coupled with single-cell epigenomic readouts to determine whether the resulting methylation changes occur as a continuous gradient (supporting the rheostat model) or as abrupt, all-or-nothing leaps (falsifying it) [49,50]. Their functions are further shaped by nuclear architecture and interactions with other epigenetic regulators, including histone-modifying enzymes and chromatin remodelers [51,52]. This integrated model provides a conceptual framework for understanding how lncRNAs may contribute to epigenetic heterogeneity and plasticity in HCC, with potential implications for tumor initiation, progression, and therapeutic response [30].

**Table 1:** Comprehensive landscape of the lncRNA–DNA methylation axis in hepatocellular carcinoma: mechanisms, targets, and evidence levels.

lncRNA	Modulator/Enzyme(s)	Mechanism of Action	Target Loci & Methylation Shift	Functional Phenotype	Cancer Type & Model System	Evidence Level *	Ref.
*HOTAIR*	DNMT1/DNMT3B	Scaffold: Bridges DNMT complexes to chromatin	Hypermethylation (Silencing) of hepatocyte-specific *miR-122*	Accelerated proliferation, delayed apoptosis	HCC (*In vitro, In vivo*, Patient cohorts)	Direct (RIP/ChIP demonstrated)	[22,23]
*GIHCG*	EZH2/DNMT1	Indirect Scaffold: Recruits DNMT1 via intermediate broad modifier PRC2	Hypermethylation (Silencing) of *miR-200b/a/429* loci	Induces EMT and promotes metastatic traits	HCC (*In vitro, In vivo*, Patient cohorts)	Indirect (Scaffolds intermediate complex)	[32]
*H19*	SAHH (Metabolic enzyme)	Metabolic Sensor: Binds SAHH, disrupting SAH/SAM metabolic ratios	Global hypomethylation (Inherent disruption of DNMT activity)	Adaptation to hypoxic stress and metabolic starvation	HCC (*In vitro*, Xenografts)	Direct (Protein binding), Indirect (DNA targeting)	[53,54,55]
*MALAT1*	Epigenetic complexes	Architectural: Shapes nuclear speckles to alter spatial distribution	Global distribution shifts of methylation machinery	Contributes to Sorafenib resistance/ferroptosis evasion	HCC (*In vitro*, Patient cohorts)	Indirect (Spatial/Nuclear architecture)	[27,28]
*NEAT1*	DNMT1	Guide/Targeting: Enhances DNMT1 focal enrichment	Hypermethylation (Silencing) of *TP53*, *cGAS*, *STING*	Malignant phenotype, immune evasion	Lung/HCC (*In vitro*, Xenografts)	Direct (Demonstrated localized recruitment)	[25]
*FTX*	DNMT1 (Targeted by)	Epigenetic Target: Victim of aberrant DNMT focal activity	Promoter hypermethylation leads to loss of *FTX*	Evasion of ferroptosis, drug-persister phenotype	HCC (*In vitro, In vivo*)	Direct (Valid substrate for DNMT1)	[56]
*lncRNA-ATB*	Epigenetic machinery (TGF-β dep.)	Signaling Node: Upregulated by extracellular cues to drive epigenetics	Altered methylation status of epithelial hubs (e.g., *CDH1*)	Drives the invasion-metastasis (EMT) cascade	HCC (*In vitro*, Xenografts, Patient cohorts)	Indirect (Signaling coordinator)	[57]
*SNHG1*	Epigenetic networks	Therapeutic Modulator: Dysregulates downstream signaling	Modulates genes within the Akt/apoptotic cascade	Epigenetically encoded resistance to Sorafenib	HCC (*In vitro*, Patient cohorts)	Indirect (Transcriptional network remodeling)	[58]
*TARID*	TET1/GADD45A	Direct Guide (R-loop dependent): Anchors TET1	Prevents *de novo* methylation/Active demethylation of TCF21	Transcriptional reactivation and tumor suppression	Breast/Solid Tumors (Mechanistic blueprint for HCC)	Direct (Validated via DRIP-seq/R-loops)	[35,36]
*MEG3*	DNMTs (TGF-β dep.)	Direct Guide (RNA:DNA Triplex dependent)	Repression of TGF-β pathway genes	EMT regulation and transcriptional plasticity	Hepatic/Solid Tumors (*In vitro*)	Direct (Sequence-specific Triplex prediction)	[40,59]

* Evidence Level Definitions: Direct: Literature provides robust biochemical evidence of physical interaction and localized targeting (e.g., RIP, RNA-ChIP, DRIP-seq for R-loops). Indirect: The lncRNA alters methylation by scaffolding broad intermediate chromatin-remodeling complexes (e.g., PRC2), modulating metabolic substrate availability (e.g., SAHH), or acting via downstream signaling networks without functioning as a direct sequence-specific nucleic acid anchor. Examples like *TARID* and *MEG3* are included as rigorously validated architectural blueprints (R-loops/triplexes) in related tumor models, providing conceptual frameworks awaiting definitive spatial epigenomic mapping in HCC. Abbreviations: HCC = Hepatocellular Carcinoma; DNMT = DNA Methyltransferase; TET = Ten-Eleven Translocation.

## 3. DNA-Methylation-Mediated Regulation of lncRNA Expression in HCC

While lncRNAs actively shape DNA methylation landscapes in HCC, accumulating evidence indicates that this regulatory relationship is bidirectional [17,60,61]. Aberrant DNA methylation also serves as a critical upstream mechanism controlling lncRNA expression, thereby establishing feedback loops that reinforce epigenetic dysregulation during hepatocarcinogenesis [62,63]. Understanding how DNA methylation modulates lncRNA transcription is essential for a comprehensive view of the lncRNA–DNA methylation axis [20].

### 3.1. Promoter-Methylation-Dependent Silencing of lncRNAs

Similar to protein-coding genes, many lncRNAs are transcribed from promoters enriched in CpG islands and are therefore susceptible to DNA-methylation-mediated repression [63,64,65]. Hypermethylation of lncRNA promoters has been frequently observed in HCC and is associated with transcriptional silencing of tumor-suppressive lncRNAs [59,62]. This epigenetic inactivation can disrupt regulatory networks involved in cell cycle control, apoptosis, and differentiation, thereby contributing to malignant transformation [63,66].

DNMT-mediated promoter methylation represents a stable and heritable mechanism for suppressing lncRNA expression [67]. Once established, these methylation marks can persist through cell division, leading to long-term silencing of protective lncRNA programs [62]. Such epigenetically silenced lncRNAs may normally function to restrain oncogenic signaling or maintain hepatocyte identity, highlighting the pathogenic significance of their repression in HCC [68,69].

### 3.2. DNA Demethylation and Reactivation of Oncogenic lncRNAs

In contrast to promoter-hypermethylation-induced silencing, hypomethylation or active demethylation can lead to aberrant activation of oncogenic lncRNAs [63]. Dysregulation of TET enzymes and global methylation instability in HCC may facilitate the demethylation of lncRNA regulatory regions, resulting in sustained overexpression [17,70]. These activated lncRNAs often participate in pathways that promote proliferation, invasion, and therapeutic resistance, thereby amplifying malignant phenotypes [71].

Importantly, demethylation-driven lncRNA activation may occur in a context-dependent manner, influenced by metabolic state, hypoxia, or oncogenic signaling [72,73,74]. This dynamic regulation enables cancer cells to rapidly adjust lncRNA expression profiles in response to environmental cues, further contributing to epigenetic plasticity and tumor heterogeneity [71].

### 3.3. Feedback Loops Within the lncRNA–DNA Methylation Network

**Figure 2:**
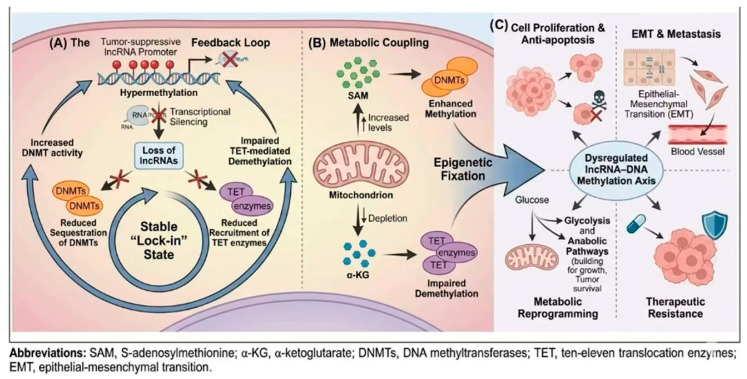
The feedback loop between lncRNA–DNA methylation and its biological impact on HCC progression. The bidirectional regulatory axis integrates metabolic cues to drive phenotypic adaptation and malignant evolution in hepatocellular carcinoma. (**A**) The Feedback Loop: Under pathological conditions, the hypermethylation of tumor-suppressive lncRNA promoters leads to transcriptional silencing. The resulting loss of these lncRNAs—which normally function to sequester DNMTs or recruit TET enzymes—further facilitates DNA hypermethylation at their own loci and downstream targets, establishing a stable, heritable “lock-in” state. (**B**) Metabolic Coupling: Liver-specific metabolic fluctuations influence the catalytic efficiency of the methylation machinery. Increased levels of S-adenosylmethionine (SAM) provide methyl donors for DNMTs, while the depletion of α-ketoglutarate (α-KG) impairs TET-mediated demethylation, collectively tilting the rheostat toward epigenetic fixation. (**C**) Biological Consequences: The dysregulated lncRNA–DNA methylation axis orchestrates diverse oncogenic phenotypes, including the pro-motion of cell proliferation and anti-apoptosis, the induction of epithelial–mesenchymal transition (EMT) for metastasis, the facilitation of metabolic reprogramming and the development of therapeutic resistance. Note: The dynamic structural sensing of lncRNAs induced by metabolic stress (such as hypoxia or α-KG/SAM fluctuations) represents a plausible theoretical framework synthesized from indirect data, which awaits definitive experimental confirmation in HCC.

The reciprocal regulation between lncRNAs and DNA methylation machinery gives rise to self-reinforcing feedback loops in HCC [17,20,63] (Figure 2). lncRNAs that are activated by DNA demethylation may subsequently recruit DNMTs or modulate TET activity to reshape methylation patterns at downstream target genes [19,75]. Conversely, lncRNAs silenced by promoter hypermethylation may normally function to restrain DNMT activity, and their loss further exacerbates methylation imbalance [56,62].

Such feedback circuitry stabilizes aberrant epigenetic states and reduces the likelihood of spontaneous reversion to normal transcriptional programs. From a systems perspective, these interconnected loops transform DNA methylation and lncRNA regulation into a tightly coupled network rather than a linear pathway, providing a mechanistic explanation for the persistence of epigenetic abnormalities in HCC [17,19,76,77].

Indeed, the lncRNA–DNA methylation axis in HCC does not operate in isolation, but is embedded within self-reinforcing feedback loops that promote epigenetic fixation. Once the promoter of a tumor-suppressive lncRNA undergoes DNMT-mediated hypermethylation, transcriptional silencing of the lncRNA ensues [62,63]. Because such lncRNAs may normally sequester DNMTs or recruit TET enzymes to facilitate local DNA demethylation, their loss further reinforces hypermethylation at their own loci as well as downstream targets, thereby establishing a stable “lock-in” state [17]. This feedback mechanism is particularly pronounced in the HCC microenvironment, where alterations in liver-specific metabolites, including α-ketoglutarate (α-KG) and S-adenosylmethionine (SAM), modulate the catalytic activities of TET dioxygenases and DNMTs, respectively [78,79]. By coupling metabolic fluctuations with lncRNA-directed epigenetic targeting, HCC cells generate an epigenetic rheostat that supports rapid phenotypic adaptation under hypoxic or nutrient-deprived conditions.

Specifically, this coupling extends beyond mere enzymatic substrate availability to actively modulate lncRNA function. A prominent example in HCC is the interplay between microenvironmental stress and the lncRNA *H19*. Under hypoxic stress, the expression of *H19* is significantly upregulated in a hypoxia-inducible factor 1-alpha (HIF-1α)-dependent manner. This metabolic–epigenetic coupling enables *H19* to structurally interact with critical chromatin modifiers and act as a dynamic sensor. By translating hypoxic starvation signals into profound alterations of the downstream epigenetic landscape, this validated mechanism provides a concrete exemplar of how HCC cells utilize lncRNAs for rapid environmental adaptation [53,54,55].

## 4. lncRNA–DNA Methylation Axis in Shaping HCC Phenotypes

While the preceding sections focus on the mechanistic interplay between lncRNAs and DNA methylation, these epigenetic interactions ultimately exert their impact through the regulation of tumor phenotypes [66,80,81]. In HCC, aberrant DNA methylation patterns coordinated by lncRNAs translate epigenetic dysregulation into functional outcomes that contribute to disease initiation, progression, and therapeutic adaptation [20,62]. Rather than acting through isolated pathways, the lncRNA–DNA methylation axis integrates multiple biological processes, enabling cancer cells to dynamically balance proliferation, invasiveness, metabolic demands, and survival under therapeutic pressure [30,82]. In this section, we summarize how lncRNA-mediated DNA methylation remodeling shapes key HCC phenotypes, including uncontrolled cell growth, epithelial–mesenchymal transition, metabolic reprogramming, tumor microenvironment modulation, and therapy resistance, thereby linking epigenetic mechanisms to biological and clinical consequences [20,83].

### 4.1. Regulation of Cell Proliferation and Apoptosis by the lncRNA–DNA Methylation Axis

Uncontrolled cell proliferation and evasion of apoptosis are fundamental hallmarks of HCC. While genetic alterations contribute to these phenotypes, accumulating evidence indicates that epigenetic reprogramming plays a decisive role in sustaining aberrant growth signals [84]. In particular, lncRNA-mediated modulation of DNA methylation has emerged as a critical mechanism linking epigenetic dysregulation to proliferative advantage and apoptotic resistance in HCC [63,81,85].

To sustain uncontrolled proliferation, the lncRNA–DNA methylation axis functionally dismantles critical cell cycle checkpoints and apoptotic cascades [60,86]. Rather than acting strictly as generic transcriptional repressors, lncRNAs direct targeted epigenetic silencing toward specific locus hubs [14]. An archetypal example in HCC is the lncRNA *HOTAIR*, which functions as an epigenetic scaffold bridging DNMT1/DNMT3B to the promoter of *miR-122*—a critical hepatocyte-specific tumor suppressor. This precise locus-targeting induces robust hypermethylation, permanently silencing *miR-122* and thereby accelerating uncontrolled hepatocyte proliferation [23]. Similarly, the stable lncRNA-directed hypermethylation of other tumor suppressor genes, such as *CDKN1A*, *CDKN2A*, and *PTEN*, represents a durable epigenetic shift that affords HCC cells a profound growth advantage and uncouples them from normal contact inhibition [87]. Importantly, this repression is often durable, highlighting the contribution of lncRNA–DNA methylation interactions to long-term epigenetic memory in tumor cells [80].

In parallel, lncRNAs can reinforce apoptotic resistance by shaping DNA methylation patterns at genes involved in intrinsic and extrinsic cell death pathways [88]. Hypermethylation-mediated silencing of pro-apoptotic regulators, including members of the BCL-2 family and death receptor signaling components, has been associated with lncRNA dysregulation in HCC [57,89,90]. By maintaining these genes in a transcriptionally repressed state, lncRNAs indirectly protect cancer cells from apoptosis induced by oncogenic stress or therapeutic intervention [91].

Beyond promoting hypermethylation, lncRNA-mediated control of DNA demethylation also contributes to proliferation and survival [92]. By modulating TET enzyme expression or chromatin localization, lncRNAs can facilitate locus-specific demethylation and reactivation of genes that support cell cycle progression, DNA replication, and survival signaling [35]. This dual capacity to repress tumor suppressors while activating growth-promoting pathways underscores the versatility of the lncRNA–DNA methylation axis in shaping proliferative phenotypes [86].

Notably, the impact of lncRNA-mediated methylation remodeling on proliferation and apoptosis is highly context-dependent [86]. The same lncRNA may exert distinct effects depending on cellular state, epigenetic background, and microenvironmental cues [93]. Such context specificity reflects the epigenetic rheostat function of lncRNAs described earlier, whereby fine-tuning rather than binary regulation of DNA methylation enables adaptive control of gene expression programs [94].

Collectively, these findings indicate that lncRNA-driven DNA methylation remodeling serves as a central epigenetic mechanism linking chromatin regulation to uncontrolled proliferation and apoptotic evasion in HCC [95]. By integrating DNMT- and TET-dependent pathways, lncRNAs provide cancer cells with a flexible yet stable means of sustaining growth-promoting transcriptional programs, thereby contributing to tumor initiation and progression [86,92].

### 4.2. Regulation of Epithelial–Mesenchymal Transition and Metastasis by the lncRNA–DNA Methylation Axis

Epithelial–mesenchymal transition (EMT) and subsequent metastatic dissemination represent critical steps in HCC progression and are closely associated with poor prognosis [96]. While previous sections highlighted typical promoter hypermethylation, resolving the EMT phenotype requires a sharp focus on its unique reversible and dynamic nature [97]. Increasing evidence indicates that lncRNA-mediated modulation of DNA methylation plays a pivotal role in orchestrating EMT and metastasis in HCC [98].

Instead of acting merely as binary on/off switches, lncRNAs function as “epigenetic rheostats” that enable partial or hybrid EMT states, balancing invasiveness with proliferative capacity [16,48]. Through recruitment of DNA methyltransferases, lncRNAs can promote hypermethylation and silencing of epithelial markers, such as *CDH1* (E-cadherin), thereby weakening cell–cell adhesion and facilitating EMT [97]. Concurrently, demethylation or hypomethylation at promoters of mesenchymal genes and EMT-inducing transcription factors, including *VIM* [99], *SNAI1* [100], and *ZEB1* [101], enhances their expression and contributes to phenotypic transition [88]. This coordinated remodeling of DNA methylation patterns enables a stable yet adaptable EMT program in HCC cells [96,102].

Furthermore, lncRNAs heavily modulate this dynamic EMT through indirect regulation of signaling pathways that converge on DNA methylation machinery [86]. For instance, aberrant activation of canonical pathways, such as TGF-β signaling, profoundly upregulates specific non-coding transcripts like *lncRNA-ATB* (lncRNA activated by TGF-β). *lncRNA-ATB* acts as a master epigenetic coordinator that silences epithelial genes and drives the invasion-metastasis cascade in HCC [57] (Table 1). Concurrently, transcriptomic dysregulation of other specific transcripts, such as the lncRNA *HULC*, further reshapes the methylation status of EMT-inducing hubs like ZEB1 [103]. This feed-forward regulatory circuitry amplifies invasive signaling and reinforces epigenetically encoded metastatic traits [102]. By acting at the interface between signaling cascades and chromatin regulation, lncRNAs translate transient extracellular cues into persistent epigenetic changes [14].

Beyond primary tumor invasion, lncRNA-mediated DNA methylation remodeling contributes to later stages of metastasis, including intravasation, survival in circulation, and colonization of distant organs [104]. Epigenetic silencing of metastasis suppressor genes and activation of genes involved in extracellular matrix remodeling, angiogenesis, and stemness have been linked to lncRNA dysregulation in HCC [16,57,105]. These methylation-dependent transcriptional programs enhance cellular plasticity and adaptability, facilitating metastatic outgrowth under diverse microenvironmental conditions [102].

In summary, rather than merely driving a unidirectional transition, the lncRNA–DNA methylation axis governs the dynamic reversibility of EMT in HCC [16,102]. This epigenetic rheostat endows cancer cells with the critical plasticity to toggle between invasive and proliferative states, ensuring successful metastatic progression across diverse microenvironments [48,96].

### 4.3. Metabolic Reprogramming and Tumor Microenvironment Shaped by the lncRNA–DNA Methylation Axis

Metabolic reprogramming and remodeling of the tumor microenvironment (TME) are increasingly recognized as key determinants of HCC progression [106,107]. Given the central role of the liver in systemic metabolism, epigenetic mechanisms that couple metabolic cues to transcriptional regulation are particularly relevant in HCC [108]. Rather than acting through generic epigenetic silencing, the lncRNA–DNA methylation axis functions as an active sensor, mediating both cancer cell–intrinsic metabolic adaptations and microenvironmental interactions that support tumor growth [17,109].

lncRNA-mediated DNA methylation remodeling has been implicated in the regulation of genes involved in glucose metabolism, lipid synthesis, and mitochondrial function [17,110]. Instead of traditional binary switches, this epigenetic regulation enables metabolic gene expression programs to be stably maintained even under fluctuating nutrient availability. This durably encodes the activation of glycolytic pathways and anabolic processes, thereby supporting the profound metabolic flexibility characteristic of HCC cells [111,112].

In parallel, this regulatory axis governs the metabolic interplay between cancer cells and the surrounding microenvironment [98,113]. Epigenetic fine-tuning of cytokines, chemokines, and extracellular matrix–modifying enzymes actively coordinates the recruitment and functional polarization of stromal and immune cells within the TME [114,115]. Consequently, lncRNA-driven inflammatory signaling reshapes immune cell infiltration, establishing an immunosuppressive symbiotic niche that shields the tumor and facilitates progression [116,117,118].

Hypoxia represents a prime microenvironmental stress in solid tumors and a potent driver of this metabolic–epigenetic coupling [53]. Specific hypoxia-responsive lncRNAs effectively translate low-oxygen signals into stable epigenetic adaptations, linking oxygen availability to heritable, adaptive gene expression programs [72,73]. This specific epigenetic memory enables HCC cells to survive and proliferate in hypoxic niches while simultaneously promoting crucial survival processes such as angiogenesis and metabolic rewiring [54,119].

Collectively, these findings highlight that the lncRNA–DNA methylation axis serves primarily as a dynamic molecular interface—translating fluctuating metabolic states and microenvironmental cues into stable epigenetic regulation [115,120]. By seamlessly integrating intrinsic metabolic demands with extrinsic signals from the TME, lncRNAs are indispensable for establishing a highly permissive, adaptive niche that supports continuous HCC progression [121].

### 4.4. Therapy Resistance and Clinical Implications of the lncRNA–DNA Methylation Axis

Therapeutic resistance remains a major obstacle in the clinical management of HCC, limiting the efficacy of systemic therapies and contributing to high recurrence rates [122]. While genetic alterations provide fixed survival traits, increasing evidence suggests that epigenetic mechanisms play a far more dynamic role by actively encoding ‘drug persister’ states in response to therapeutic stress [123,124]. In particular, lncRNA-mediated DNA methylation remodeling serves as a central engine driving this durable therapy resistance in HCC [83].

A defining feature of this mechanism is the lncRNA-driven epigenetic lockdown of genes associated with drug sensitivity and apoptotic response [58]. Rather than acting via transient transcriptional repression, lncRNAs induce stable promoter hypermethylation of targeted tumor suppressors [23], thereby profoundly eroding the cytotoxic efficacy of therapies [58]. For instance, the lncRNA *SNHG1* actively contributes to acquired resistance against targeted therapies, such as Sorafenib, by remodeling epigenetic networks that govern Akt signaling and apoptotic responses [58]. Furthermore, epigenetic plasticity allows HCC cells to evade alternative cell death modalities. A prime example is the DNMT1-dependent hypermethylation and subsequent silencing of the tumor-suppressive lncRNA *FTX*, an epigenetic suppression that uncouples HCC cells from ferroptosis, thereby generating a highly resilient, drug-persister phenotype [56] (Table 1). Such epigenetically encoded resistance programs may persist even after drug withdrawal, establishing a uniquely durable form of long-term therapeutic adaptation [115].

In parallel, lncRNAs actively confer survival advantages by fine-tuning DNA demethylation pathways. Coordinated alterations in demethylation dynamics have been deeply implicated in resurrecting silenced genes linked to stemness and therapeutic evasion [125]. By selectively reprogramming these epigenetic networks under selective therapeutic pressure, lncRNAs facilitate rapid phenotypic shifts [86]. This highly coordinated adaptability is particularly lethal in HCC, as it continuously generates intratumoral heterogeneity and epigenetic plasticity that undermine durable treatment responses [126].

Beyond tumor cell–intrinsic mechanisms, the lncRNA–DNA methylation axis uniquely extends its protective effects by co-opting the tumor microenvironment [127]. Epigenetic rewiring of cytokine arrays, immune checkpoint molecules, and angiogenic factors actively reshapes immune surveillance to create a hostile, drug-resistant niche [128,129]. Such dynamic microenvironmental modulation further complicates therapeutic targeting, highlighting that epigenetic resistance in HCC is an orchestrated, multicellular phenomenon [130].

From a clinical perspective, acknowledging this plasticity suggests that components of the lncRNA–DNA methylation network hold substantial promise as predictive biomarkers for disease recurrence [86]. However, translating these insights into clinical practice remains challenging [115]. The context-dependent and pleiotropic functions of lncRNAs, as well as the global effects of DNA methylation–targeting agents, necessitate careful evaluation of specificity, safety, and patient stratification [128]. Future therapeutic strategies must evolve to integrate context-aware epigenetic modulators with existing treatments, a paradigm shift that requires extensive preclinical and clinical validation to effectively overcome HCC resistance [130].

## 5. Discussion

Accumulating evidence underscores the central role of epigenetic dysregulation in HCC, with DNA methylation emerging as a key determinant of transcriptional plasticity and tumor heterogeneity [120,131]. In this review, we synthesize current findings to delineate the lncRNA–DNA methylation axis as a dynamic and bidirectional regulatory network that integrates chromatin modification, nuclear organization, and gene expression control in HCC [20].

Rather than acting as passive transcriptional byproducts, lncRNAs have emerged as active coordinators of DNA methylation remodeling [14]. Through scaffold-based assembly, guide-like targeting, and modulation of nuclear architecture, lncRNAs can confer locus specificity and adaptability to DNMT- and TET-mediated processes, partially overcoming the intrinsic lack of sequence specificity of DNA methylation enzymes [132,133]. Conversely, DNA methylation serves as an upstream regulator of lncRNA expression, establishing feedback loops that stabilize aberrant epigenetic states [81]. Together, these reciprocal interactions support a conceptual model in which lncRNAs act as epigenetic rheostats that fine-tune DNA methylation landscapes in a context-dependent manner [81,134].

The functional consequences of this regulatory axis extend beyond epigenetic alterations per se to shape core tumor-associated phenotypes, including uncontrolled proliferation, apoptotic resistance, epithelial–mesenchymal transition, metabolic reprogramming, tumor microenvironment remodeling, and therapeutic resistance [20]. By linking epigenetic mechanisms to biological outcomes, the lncRNA–DNA methylation network provides a unifying framework for understanding how epigenetic plasticity supports HCC initiation, progression, and adaptation to therapeutic pressure. Notably, these effects are rarely driven by individual lncRNAs in isolation but instead arise from coordinated, network-level remodeling of DNA methylation states across multiple regulatory nodes.

Crucially, DNA-methylation-dependent regulation of lncRNA expression contributes significantly to intertumoral and intratumoral heterogeneity in HCC [63]. Differential methylation patterns across tumors or within distinct cellular subpopulations can generate diverse lncRNA expression profiles, leading to variability in downstream regulatory networks [82,135]. This heterogeneity may underlie differences in disease aggressiveness, metastatic potential, and therapeutic response among patients [20].

Despite these advances, several challenges remain. The context-dependent and often pleiotropic functions of lncRNAs complicate efforts to define their precise roles across different stages of disease and cellular subpopulations [136]. Moreover, the global nature of DNA methylation–targeting strategies raises concerns regarding specificity and unintended epigenetic consequences [137]. Future studies integrating single-cell and spatial epigenomics, CRISPR-based epigenetic perturbation and longitudinal modeling will be essential to resolve these complexities and to establish causal relationships within lncRNA–DNA methylation regulatory networks [138,139].

From a translational perspective, components of the lncRNA–DNA methylation axis hold substantial promise as predictive biomarkers for disease stratification and therapeutic response in HCC. Given that aberrant DNA methylation signatures and certain lncRNAs can be detected in circulating cell-free DNA/RNA or exosomes, their non-invasive biomarker feasibility is increasingly becoming a clinical reality [115]. However, resolving the “druggability” of this axis presents distinct challenges. Direct targeting of individual lncRNAs or global DNA methylation regulators (e.g., using broad-spectrum DNMT inhibitors like decitabine) remains challenging due to poor specificity and profound off-target epigenetic toxicities [71]. To bypass these limitations, the field is rapidly advancing toward sequence-specific interventions. A highly promising frontier is CRISPR/dCas9-mediated epigenetic editing. By fusing an inactive Cas9 to the catalytic domains of DNMTs or TETs, researchers can achieve programmable, locus-specific methylation rewriting guided by engineered sgRNAs—offering a precision alternative to system-wide methylation inhibition [137,138]. Concurrently, directly depleting oncogenic lncRNAs using therapeutic antisense oligonucleotides (ASOs) or siRNAs is gaining momentum. While systematic delivery remains a major constraint in most solid tumors, HCC presents a unique pharmacological advantage. The liver’s natural filtering capacity, combined with the advent of lipid nanoparticle (LNP) delivery systems and GalNAc-conjugated oligonucleotides, specifically circumvents these delivery constraints, enabling highly localized hepatic targeting.

Finally, navigating the clinical trial landscape of epigenetic therapies in HCC requires a combinatorial approach. While early monotherapy trials of epigenetic modifiers in solid tumors have shown modest success, emerging clinical strategies now focus on synergistic regimens. Future precision oncology trials must robustly assess whether targeting specific node-hub lncRNAs or their downstream methylation networks can successfully reprogram the hostile tumor microenvironment, thereby collateral-sensitizing HCC to existing first-line agents such as Sorafenib, Lenvatinib, or immune checkpoint inhibitors [17,140].

## 6. Conclusions

In conclusion, viewing DNA methylation regulation through the lens of lncRNA-mediated coordination provides a conceptual framework that reconciles epigenetic stability with plasticity in HCC. By emphasizing mechanistic integration over descriptive cataloging, this perspective advances our understanding of hepatocarcinogenesis and highlights future directions for epigenetic research and therapeutic development.

## Data Availability

No new data were created or analyzed in this study. Data sharing is not applicable to this article.

## Abbreviations

The following abbreviations are used in this manuscript:

HCC	Hepatocellular Carcinoma
lncRNA	Long Non-coding RNA
DNMT	DNA Methyltransferase
TET	Ten-Eleven Translocation
EMT	Epithelial–Mesenchymal Transition

## References

[1] Harris P.S., Hansen R.M., Gray M.E., Massoud O.I., McGuire B.M., Shoreibah M.G. (2019). Hepatocellular carcinoma surveillance: An evidence-based approach. World J. Gastroenterol..

[2] Guo D.Z., Zhang X., Zhang S.Q., Zhang S.Y., Zhang X.Y., Yan J.Y., Dong S.Y., Zhu K., Yang X.R., Fan J. (2024). Single-cell tumor heterogeneity landscape of hepatocellular carcinoma: Unraveling the pro-metastatic subtype and its interaction loop with fibroblasts. Mol. Cancer.

[3] Hogg S.J., Beavis P.A., Dawson M.A., Johnstone R.W. (2020). Targeting the epigenetic regulation of antitumour immunity. Nat. Rev. Drug Discov..

[4] Skvortsova K., Iovino N., Bogdanović O. (2018). Functions and mechanisms of epigenetic inheritance in animals. Nat. Rev. Mol. Cell Biol..

[5] Wagner W. (2025). Epigenetic networks coordinate DNA methylation across the genome. Mol. Ther..

[6] Nishiyama A., Nakanishi M. (2021). Navigating the DNA methylation landscape of cancer. Trends Genet..

[7] Shi J., Xu J., Chen Y.E., Li J.S., Cui Y., Shen L., Li J.J., Li W. (2021). The concurrence of DNA methylation and demethylation is associated with transcription regulation. Nat. Commun..

[8] Dai H.Q., Wang B.A., Yang L., Chen J.J., Zhu G.C., Sun M.L., Ge H., Wang R., Chapman D.L., Tang F. (2016). TET-mediated DNA demethylation controls gastrulation by regulating Lefty-Nodal signalling. Nature.

[9] Jyotishi C., Prajapati S., Patel M., Gupta R. (2025). Exploring Single-Cell and Multi-Omics Technologies and Their Role in Unravelling Tumor Heterogeneity of Hepatocellular Carcinoma. J. Liver Cancer.

[10] Zhou P.Y., Zhou C., Gan W., Tang Z., Sun B.Y., Huang J.L., Liu G., Liu W.R., Tian M.X., Jiang X.F. (2023). Single-cell and spatial architecture of primary liver cancer. Commun. Biol..

[11] Herman A.B., Tsitsipatis D., Gorospe M. (2022). Integrated lncRNA function upon genomic and epigenomic regulation. Mol. Cell.

[12] Peng W.X., Koirala P., Mo Y.Y. (2017). LncRNA-mediated regulation of cell signaling in cancer. Oncogene.

[13] dos Santos D.B., Fernandez G.J., Silva L.T., Silva G.F., Lima E.O., Galvani A.F., Pereira G.L., Ferrasi A.C. (2025). lncRNAs as Biomarkers of Hepatocellular Carcinoma Risk and Liver Damage in Advanced Chronic Hepatitis C. Curr. Issues Mol. Biol..

[14] Rinn J.L., Chang H.Y. (2012). Genome Regulation by Long Noncoding RNAs. Annu. Rev. Biochem..

[15] Yao R.W., Wang Y., Chen L.L. (2019). Cellular functions of long noncoding RNAs. Nat. Cell Biol..

[16] Wang K.C., Chang H.Y. (2011). Molecular Mechanisms of Long Noncoding RNAs. Mol. Cell.

[17] Huang W., Li H., Yu Q., Xiao W., Wang D.O. (2022). LncRNA-mediated DNA methylation: An emerging mechanism in cancer and beyond. J. Exp. Clin. Cancer Res..

[18] Leisegang M.S., Warwick T., Stötzel J., Brandes R.P. (2024). RNA-DNA triplexes: Molecular mechanisms and functional relevance. Trends Biochem. Sci..

[19] Yang Z., Xu F., Teschendorff A.E., Zhao Y., Yao L., Li J., He Y. (2022). Insights into the role of long non-coding RNAs in DNA methylation mediated transcriptional regulation. Front. Mol. Biosci..

[20] Shi Z., Jin S., Liu X., Jiang M., Fang Y., Khadaroo P.A., Lin H., Fan X. (2025). The epigenetic regulatory network of long noncoding RNAs in hepatocellular carcinoma. Genes. Dis..

[21] Zhao Y., Sun H., Wang H. (2016). Long noncoding RNAs in DNA methylation: New players stepping into the old game. Cell Biosci..

[22] Hsu C.Y., Jamal A., Kamal M.A., Ahmad F., Bokov D.O., Mustafa Y.F., Saud A., Kulsum S.N., Jawad M.A., Gabble B.C. (2025). Pathological roles of lncRNA *HOTAIR* in liver cancer: An updated review. Gene.

[23] Cheng D., Deng J.G., Zhang B., He X.Y., Meng Z., Li G.L., Ye H.L., Zheng S.Y., Wei L.S., Deng X.G. (2018). LncRNA *HOTAIR* epigenetically suppresses *miR-122* expression in hepatocellular carcinoma via DNA methylation. eBioMedicine.

[24] Kotake Y., Nakagawa T., Kitagawa K., Suzuki S., Liu N., Kitagawa M., Xiong Y. (2011). Long non-coding RNA *ANRIL* is required for the PRC2 recruitment to and silencing of *p15(INK4B)* tumor suppressor gene. Oncogene.

[25] Ma F., Lei Y.Y., Ding M.G., Luo L.H., Xie Y.C., Liu X.L. (2020). LncRNA *NEAT1* Interacted With DNMT1 to Regulate Malignant Phenotype of Cancer Cell and Cytotoxic T Cell Infiltration via Epigenetic Inhibition of *p53*, *cGAS*, and *STING* in Lung Cancer. Front. Genet..

[26] Shin T.J., Lee K.H., Cho J.Y. (2020). Epigenetic Mechanisms of LncRNAs Binding to Protein in Carcinogenesis. Cancers.

[27] Shi C.J., Pang F.X., Lei Y.H., Deng L.Q., Pan F.Z., Liang Z.Q., Xie T., Wu X.L., Wang Y.Y., Xian Y.F. (2025). 5-methylcytosine methylation of *MALAT1* promotes resistance to sorafenib in hepatocellular carcinoma through ELAVL1/SLC7A11-mediated ferroptosis. Drug Resist. Updat..

[28] Bocchetti M., Cossu A.M., Porru M., Ferraro M.G., Irace C., Tufano R., Vitale G., Misso G., Amodio N., Scrima M. (2025). *MiR-423-5p* is a metabolic and growth tuner in hepatocellular carcinoma via *MALAT-1* and mitochondrial interaction. J. Exp. Clin. Cancer Res..

[29] Nazih M., Waked I., Abdelsattar S., Al-Amodi H.S., Kamel H.F.M., Attia M.M., Khoder A.I., Hassan S.B., Mahmoud Abdel-Latif M. (2026). Pharmacogenomics of Sorafenib in Hepatocellular Carcinoma (HCC): A LncRNA-Expression Guided Approach Using *UCA1* and *MALAT1* for Personalizing Therapy in a 154-Patient Cohort. Pharmaceuticals.

[30] Statello L., Guo C.J., Chen L.L., Huarte M. (2021). Gene regulation by long non-coding RNAs and its biological functions. Nat. Rev. Mol. Cell Biol..

[31] Davidovich C., Cech T.R. (2015). The recruitment of chromatin modifiers by long noncoding RNAs: Lessons from PRC2. RNA.

[32] Sui C.J., Zhou Y.M., Shen W.F., Dai B.H., Lu J.J., Zhang M.F., Yang J.M. (2016). Long noncoding RNA *GIHCG* promotes hepatocellular carcinoma progression through epigenetically regulating *miR-200b/a/429*. J. Mol. Med..

[33] López-Moyado I.F., Ko M., Hogan P.G., Rao A. (2024). TET Enzymes in the Immune System: From DNA Demethylation to Immunotherapy, Inflammation, and Cancer. Annu. Rev. Immunol..

[34] Zhang X., Zhang Y., Wang C., Wang X. (2023). TET (Ten-eleven translocation) family proteins: Structure, biological functions and applications. Signal Transduct. Target. Ther..

[35] Arab K., Park Y.J., Lindroth A.M., Schäfer A., Oakes C., Weichenhan D., Lukanova A., Lundin E., Risch A., Meister M. (2014). Long noncoding RNA *TARID* directs demethylation and activation of the tumor suppressor *TCF21* via GADD45A. Mol. Cell.

[36] Arab K., Karaulanov E., Musheev M., Trnka P., Schäfer A., Grummt I., Niehrs C. (2019). GADD45A binds R-loops and recruits TET1 to CpG island promoters. Nat. Genet..

[37] Wang L., Zhao Y., Bao X., Zhu X., Kwok Y.K., Sun K., Chen X., Huang Y., Jauch R., Esteban M.A. (2015). LncRNA Dum interacts with Dnmts to regulate Dppa2 expression during myogenic differentiation and muscle regeneration. Cell Res..

[38] Xin X., Wu M., Meng Q., Wang C., Lu Y., Yang Y., Li X., Zheng Q., Pu H., Gui X. (2018). Long noncoding RNA *HULC* accelerates liver cancer by inhibiting PTEN via autophagy cooperation to *miR15a*. Mol. Cancer.

[39] Li F., Zafar A., Luo L., Denning A.M., Gu J., Bennett A., Yuan F., Zhang Y. (2023). R-Loops in Genome Instability and Cancer. Cancers.

[40] Mondal T., Subhash S., Vaid R., Enroth S., Uday S., Reinius B., Mitra S., Mohammed A., James A.R., Hoberg E. (2015). *MEG3* long noncoding RNA regulates the TGF-β pathway genes through formation of RNA-DNA triplex structures. Nat. Commun..

[41] Chédin F. (2016). Nascent Connections: R-Loops and Chromatin Patterning. Trends Genet..

[42] Ginno P.A., Lott P.L., Christensen H.C., Korf I., Chédin F. (2012). R-loop formation is a distinctive characteristic of unmethylated human CpG island promoters. Mol. Cell.

[43] Li X., Zhou B., Chen L., Gou L.T., Li H., Fu X.D. (2017). GRID-seq reveals the global RNA-chromatin interactome. Nat. Biotechnol..

[44] Crossley M.P., Bocek M., Cimprich K.A. (2019). R-Loops as Cellular Regulators and Genomic Threats. Mol. Cell.

[45] Tani H. (2025). Biomolecules Interacting with Long Noncoding RNAs. Biology.

[46] Kelsey G., Stegle O., Reik W. (2017). Single-cell epigenomics: Recording the past and predicting the future. Science.

[47] Pastushenko I., Blanpain C. (2019). EMT Transition States during Tumor Progression and Metastasis. Trends Cell Biol..

[48] Jolly M.K., Somarelli J.A., Sheth M., Biddle A., Tripathi S.C., Armstrong A.J., Hanash S.M., Bapat S.A., Rangarajan A., Levine H. (2019). Hybrid epithelial/mesenchymal phenotypes promote metastasis and therapy resistance across carcinomas. Pharmacol. Ther..

[49] Gao Y., Xiong X., Wong S., Charles E.J., Lim W.A., Qi L.S. (2016). Complex transcriptional modulation with orthogonal and inducible dCas9 regulators. Nat. Methods.

[50] Nakamura M., Gao Y., Dominguez A.A., Qi L.S. (2021). CRISPR technologies for precise epigenome editing. Nat. Cell Biol..

[51] Kumar S., Kundu S., Sharawat S.K., Sharma A. (2025). Rewiring cancer epigenome: LncRNA as modulator of chromatin architecture and neoplastic transformation. Mamm. Genome.

[52] Begolli R., Sideris N., Giakountis A. (2019). LncRNAs as Chromatin Regulators in Cancer: From Molecular Function to Clinical Potential. Cancers.

[53] Tsai Y.P., Wu K.J. (2014). Epigenetic regulation of hypoxia-responsive gene expression: Focusing on chromatin and DNA modifications. Int. J. Cancer.

[54] Chen C., Lou T. (2017). Hypoxia inducible factors in hepatocellular carcinoma. Oncotarget.

[55] Matouk I.J., DeGroot N., Mezan S., Ayesh S., Abu-lail R., Hochberg A., Galun E. (2007). The *H19* non-coding RNA is essential for human tumor growth. PLoS ONE.

[56] Fan S., Shao C., Jia S., Xie D., Yu B. (2025). DNMT1-Dependent DNA Methylation of lncRNA *FTX* Inhibits the Ferroptosis of Hepatocellular Carcinoma. Crit. Rev. Eukaryot. Gene Expr..

[57] Yuan J.H., Yang F., Wang F., Ma J.Z., Guo Y.J., Tao Q.F., Liu F., Pan W., Wang T.T., Zhou C.C. (2014). A long noncoding RNA activated by TGF-β promotes the invasion-metastasis cascade in hepatocellular carcinoma. Cancer Cell.

[58] Li W., Dong X., He C., Tan G., Li Z., Zhai B., Feng J., Jiang X., Liu C., Jiang H. (2019). LncRNA *SNHG1* contributes to sorafenib resistance by activating the Akt pathway and is positively regulated by *miR-21* in hepatocellular carcinoma cells. J. Exp. Clin. Cancer Res..

[59] Braconi C., Kogure T., Valeri N., Huang N., Nuovo G., Costinean S., Negrini M., Miotto E., Croce C.M., Patel T. (2011). microRNA-29 can regulate expression of the long non-coding RNA gene *MEG3* in hepatocellular cancer. Oncogene.

[60] Di Ruscio A., Ebralidze A.K., Benoukraf T., Amabile G., Goff L.A., Terragni J., Figueroa M.E., De Figueiredo Pontes L.L., Alberich-Jorda M., Zhang P. (2013). DNMT1-interacting RNAs block gene-specific DNA methylation. Nature.

[61] Lim L.J., Wong S.Y.S., Huang F., Lim S., Chong S.S., Ooi L.L., Kon O.L., Lee C.G. (2019). Roles and Regulation of Long Noncoding RNAs in Hepatocellular Carcinoma. Cancer Res..

[62] Recalde M., Gárate-Rascón M., Herranz J.M., Elizalde M., Azkona M., Unfried J.P., Boix L., Reig M., Sangro B., Fernández-Barrena M.G. (2022). DNA Methylation Regulates a Set of Long Non-Coding RNAs Compromising Hepatic Identity during Hepatocarcinogenesis. Cancers.

[63] Shi Z., Liu X., Li D., Yang J., Chen H., Lin H., Fan X. (2025). Deciphering the methylation landscape of long noncoding RNAs in hepatocellular carcinoma: A focus on *LINC00942*. Clin. Epigenet..

[64] Illingworth R.S., Bird A.P. (2009). CpG islands—‘A rough guide’. FEBS Lett..

[65] Derrien T., Johnson R., Bussotti G., Tanzer A., Djebali S., Tilgner H., Guernec G., Martin D., Merkel A., Knowles D.G. (2012). The GENCODE v7 catalog of human long noncoding RNAs: Analysis of their gene structure, evolution, and expression. Genome Res..

[66] Baylin S.B., Jones P.A. (2016). Epigenetic Determinants of Cancer. Cold Spring Harb. Perspect. Biol..

[67] Kumegawa K., Maruyama R., Yamamoto E., Ashida M., Kitajima H., Tsuyada A., Niinuma T., Kai M., Yamano H.O., Sugai T. (2016). A genomic screen for long noncoding RNA genes epigenetically silenced by aberrant DNA methylation in colorectal cancer. Sci. Rep..

[68] Tsai K.W., Tsai C.Y., Chou N.H., Wang K.C., Kang C.H., Li S.C., Lao Y.H., Chang H.T. (2019). Aberrant DNA Hypermethylation Silenced LncRNA Expression in Gastric Cancer. Anticancer Res..

[69] Gaggi G., Hausman C., Cho S., Badalamenti B.C., Trinh B.Q., Di Ruscio A., Ummarino S. (2025). LncRNAs Ride the Storm of Epigenetic Marks. Genes.

[70] Kang W., Wang Q., Dai Y., Wang H., Wang M., Wang J., Zhang D., Sun P., Qi T., Jin X. (2020). Hypomethylation of *PlncRNA-1* promoter enhances bladder cancer progression through the *miR-136-5p/Smad3* axis. Cell Death Dis..

[71] Nadhan R., Isidoro C., Song Y.S., Dhanasekaran D.N. (2024). LncRNAs and the cancer epigenome: Mechanisms and therapeutic potential. Cancer Lett..

[72] Peng P.H., Hsu K.W., Chieh-Yu Lai J., Wu K.J. (2021). The role of hypoxia-induced long noncoding RNAs (lncRNAs) in tumorigenesis and metastasis. Biomed. J..

[73] Kuriakose B.B., Hjazi A., Saleh R.O., Bishoyi A.K., Jyothi S.R., Almalki S.G., Sridevi G., Chaudhary K., Zwamel A.H., Matchonov O. (2025). LncRNAs in hypoxic microenvironment; insight in their impact in cancer biology. Funct. Integr. Genom..

[74] Fang K., Xu H., Yuan S., Li X., Chen X., Fan X., Gao X., Zhang L., Sun S., Zhu X. (2024). LncRNA mediated metabolic reprogramming: The chief culprits of solid tumor malignant progression: An update review. Nutr. Metab..

[75] Yu Y., Lu X., Yan Y., Wang Y., Meng J., Tian S., Mu J. (2022). The lncRNA *KIF9-AS1* Accelerates Hepatocellular Carcinoma Growth by Recruiting DNMT1 to Promote *RAI2* DNA Methylation. J. Oncol..

[76] Ranjbar M., Heydarzadeh S., Shekari Khaniani M., Foruzandeh Z., Seif F., Pornour M., Rahmanpour D., Tarhriz V., Alivand M. (2023). Mutual interaction of lncRNAs and epigenetics: Focusing on cancer. Egypt. J. Med. Hum. Genet..

[77] Dai C., Qianjiang H., Fu R., Yang H., Shi A., Luo H. (2025). Epigenetic and epitranscriptomic role of lncRNA in carcinogenesis (Review). Int. J. Oncol..

[78] Chen C., Wang Z., Qin Y. (2022). Connections between metabolism and epigenetics: Mechanisms and novel anti-cancer strategy. Front. Pharmacol..

[79] Xu H., Zhao X., Yun Y., Gao Y., Bo C., Song L., Bai C., Yang L., Li G., Su G. (2025). Regulation of Mitochondrial Metabolism by Mfn1 Gene Encoding Mitofusin Affects Cellular Proliferation and Histone Modification. Cells.

[80] Feinberg A.P., Koldobskiy M.A., Göndör A. (2016). Epigenetic modulators, modifiers and mediators in cancer aetiology and progression. Nat. Rev. Genet..

[81] Yuan S.X., Zhang J., Xu Q.G., Yang Y., Zhou W.P. (2016). Long noncoding RNA, the methylation of genomic elements and their emerging crosstalk in hepatocellular carcinoma. Cancer Lett..

[82] Lin D.C., Mayakonda A., Dinh H.Q., Huang P., Lin L., Liu X., Ding L.W., Wang J., Berman B.P., Song E.W. (2017). Genomic and Epigenomic Heterogeneity of Hepatocellular Carcinoma. Cancer Res..

[83] Su L., Bu J., Yu J., Jin M., Meng G., Zhu X. (2024). Comprehensive review and updated analysis of DNA methylation in hepatocellular carcinoma: From basic research to clinical application. Clin. Transl. Med..

[84] Yu K., Jin Y., Zhou Y., Xu Q. (2025). Long non-coding RNA in hepatocellular carcinoma: Mechanistic insights and therapeutic perspectives. Cell. Oncol..

[85] Wang J., Liu Z.X., Huang Z.H., Wen J., Rao Z.Z. (2025). Long non-coding RNA in the regulation of cell death in hepatocellular carcinoma. World J. Clin. Oncol..

[86] Schmitt A.M., Chang H.Y. (2016). Long Noncoding RNAs in Cancer Pathways. Cancer Cell.

[87] Nagaraju G.P., Dariya B., Kasa P., Peela S., El-Rayes B.F. (2022). Epigenetics in hepatocellular carcinoma. Semin. Cancer Biol..

[88] Esteller M. (2008). Epigenetics in cancer. N. Engl. J. Med..

[89] Esteller M. (2005). Aberrant DNA methylation as a cancer-inducing mechanism. Annu. Rev. Pharmacol. Toxicol..

[90] He Y., Meng X.M., Huang C., Wu B.M., Zhang L., Lv X.W., Li J. (2014). Long noncoding RNAs: Novel insights into hepatocelluar carcinoma. Cancer Lett..

[91] Baylin S.B., Jones P.A. (2011). A decade of exploring the cancer epigenome—Biological and translational implications. Nat. Rev. Cancer.

[92] Wu X., Zhang Y. (2017). TET-mediated active DNA demethylation: Mechanism, function and beyond. Nat. Rev. Genet..

[93] Quinn J.J., Chang H.Y. (2016). Unique features of long non-coding RNA biogenesis and function. Nat. Rev. Genet..

[94] Feinberg A.P. (2014). Epigenetic stochasticity, nuclear structure and cancer: The implications for medicine. J. Intern. Med..

[95] Craig A.J., von Felden J., Garcia-Lezana T., Sarcognato S., Villanueva A. (2020). Tumour evolution in hepatocellular carcinoma. Nat. Rev. Gastroenterol. Hepatol..

[96] Nieto M.A., Huang R.Y., Jackson R.A., Thiery J.P. (2016). EMT: 2016. Cell.

[97] Tam W.L., Weinberg R.A. (2013). The epigenetics of epithelial-mesenchymal plasticity in cancer. Nat. Med..

[98] Klingenberg M., Matsuda A., Diederichs S., Patel T. (2017). Non-coding RNA in hepatocellular carcinoma: Mechanisms, biomarkers and therapeutic targets. J. Hepatol..

[99] Huber M.A., Kraut N., Beug H. (2005). Molecular requirements for epithelial-mesenchymal transition during tumor progression. Curr. Opin. Cell Biol..

[100] Peinado H., Ballestar E., Esteller M., Cano A. (2004). Snail mediates E-cadherin repression by the recruitment of the Sin3A/histone deacetylase 1 (HDAC1)/HDAC2 complex. Mol. Cell Biol..

[101] Aigner K., Dampier B., Descovich L., Mikula M., Sultan A., Schreiber M., Mikulits W., Brabletz T., Strand D., Obrist P. (2007). The transcription factor ZEB1 (deltaEF1) promotes tumour cell dedifferentiation by repressing master regulators of epithelial polarity. Oncogene.

[102] McDonald O.G., Wu H., Timp W., Doi A., Feinberg A.P. (2011). Genome-scale epigenetic reprogramming during epithelial-to-mesenchymal transition. Nat. Struct. Mol. Biol..

[103] Li S.P., Xu H.X., Yu Y., He J.D., Wang Z., Xu Y.J., Wang C.Y., Zhang H.M., Zhang R.X., Zhang J.J. (2016). LncRNA *HULC* enhances epithelial-mesenchymal transition to promote tumorigenesis and metastasis of hepatocellular carcinoma via the *miR-200a-3p/ZEB1* signaling pathway. Oncotarget.

[104] Lambert A.W., Pattabiraman D.R., Weinberg R.A. (2017). Emerging Biological Principles of Metastasis. Cell.

[105] Puisieux A., Brabletz T., Caramel J. (2014). Oncogenic roles of EMT-inducing transcription factors. Nat. Cell Biol..

[106] Pavlova N.N., Thompson C.B. (2016). The Emerging Hallmarks of Cancer Metabolism. Cell Metab..

[107] Vogel A., Cervantes A., Chau I., Daniele B., Llovet J.M., Meyer T., Nault J.C., Neumann U., Ricke J., Sangro B. (2018). Hepatocellular carcinoma: ESMO Clinical Practice Guidelines for diagnosis, treatment and follow-up. Ann. Oncol..

[108] Bueloni B., Garcia Fernandez de Barrena M., Avila M.A., Bayo J., Mazzolini G. (2025). Epigenetic mechanisms involved in hepatocellular carcinoma development and progression. eGastroenterology.

[109] Liao W., Du J., Wang Z., Feng Q., Liao M., Liu H., Yuan K., Zeng Y. (2022). The role and mechanism of noncoding RNAs in regulation of metabolic reprogramming in hepatocellular carcinoma. Int. J. Cancer.

[110] Lin W., Zhou Q., Wang C.Q., Zhu L., Bi C., Zhang S., Wang X., Jin H. (2020). LncRNAs regulate metabolism in cancer. Int. J. Biol. Sci..

[111] Scumaci D., Zheng Q. (2023). Epigenetic meets metabolism: Novel vulnerabilities to fight cancer. Cell Commun. Signal.

[112] Xu X., Peng Q., Jiang X., Tan S., Yang Y., Yang W., Han Y., Chen Y., Oyang L., Lin J. (2023). Metabolic reprogramming and epigenetic modifications in cancer: From the impacts and mechanisms to the treatment potential. Exp. Mol. Med..

[113] Perez-Medina M., Benito-Lopez J.J., Aguilar-Cazares D., Lopez-Gonzalez J.S. (2025). A Comprehensive Review of Long Non-Coding RNAs in the Cancer–Immunity Cycle: Mechanisms and Therapeutic Implications. Int. J. Mol. Sci..

[114] Jones P.A., Issa J.P., Baylin S. (2016). Targeting the cancer epigenome for therapy. Nat. Rev. Genet..

[115] Hardy T., Mann D.A. (2016). Epigenetics in liver disease: From biology to therapeutics. Gut.

[116] Denaro N., Merlano M.C., Lo Nigro C. (2019). Long noncoding RNAs as regulators of cancer immunity. Mol. Oncol..

[117] Pi Y.N., Qi W.C., Xia B.R., Lou G., Jin W.L. (2021). Long Non-Coding RNAs in the Tumor Immune Microenvironment: Biological Properties and Therapeutic Potential. Front. Immunol..

[118] Zhan D.T., Xian H.C. (2023). Exploring the regulatory role of lncRNA in cancer immunity. Front. Oncol..

[119] Li T., Mao C., Wang X., Shi Y., Tao Y. (2020). Epigenetic crosstalk between hypoxia and tumor driven by HIF regulation. J. Exp. Clin. Cancer Res..

[120] Nakamura M., Chiba T., Kanayama K., Kanzaki H., Saito T., Kusakabe Y., Kato N. (2019). Epigenetic dysregulation in hepatocellular carcinoma: An up-to-date review. Hepatol. Res..

[121] Wang F., Malnassy G., Qiu W. (2021). The Epigenetic Regulation of Microenvironment in Hepatocellular Carcinoma. Front. Oncol..

[122] Oura K., Morishita A., Hamaya S., Fujita K., Masaki T. (2023). The Roles of Epigenetic Regulation and the Tumor Microenvironment in the Mechanism of Resistance to Systemic Therapy in Hepatocellular Carcinoma. Int. J. Mol. Sci..

[123] Qi D., Qin Y., Zhu H., Li Y., Han S. (2025). Resistance of first-line targeted drugs in hepatocellular carcinoma: The epigenetic regulation mechanisms. Cell Death Dis..

[124] Su X., Li Y., Ren Y., Cao M., Yang G., Luo J., Hu Z., Deng H., Deng M., Liu B. (2024). A new strategy for overcoming drug resistance in liver cancer: Epigenetic regulation. Biomed. Pharmacother..

[125] Rasmussen K.D., Helin K. (2016). Role of TET enzymes in DNA methylation, development, and cancer. Genes. Dev..

[126] Llovet J.M., Zucman-Rossi J., Pikarsky E., Sangro B., Schwartz M., Sherman M., Gores G. (2016). Hepatocellular carcinoma. Nat. Rev. Dis. Primers.

[127] Binnewies M., Roberts E.W., Kersten K., Chan V., Fearon D.F., Merad M., Coussens L.M., Gabrilovich D.I., Ostrand-Rosenberg S., Hedrick C.C. (2018). Understanding the tumor immune microenvironment (TIME) for effective therapy. Nat. Med..

[128] Jones P.A., Ohtani H., Chakravarthy A., De Carvalho D.D. (2019). Epigenetic therapy in immune-oncology. Nat. Rev. Cancer.

[129] Cao J., Yan Q. (2020). Cancer Epigenetics, Tumor Immunity, and Immunotherapy. Trends Cancer.

[130] Housman G., Byler S., Heerboth S., Lapinska K., Longacre M., Snyder N., Sarkar S. (2014). Drug resistance in cancer: An overview. Cancers.

[131] Wali A.F., Ansari A.R., Mir P.A., El-Tanani M., Babiker R., Hussain M.S., Uppal J., Zargar A.I., Mir R.H. (2025). Epigenetic Alterations in Hepatocellular Carcinoma: Mechanisms, Biomarkers, and Therapeutic Implications. Pharmaceuticals.

[132] Schmitz S.U., Grote P., Herrmann B.G. (2016). Mechanisms of long noncoding RNA function in development and disease. Cell. Mol. Life Sci..

[133] Smith Z.D., Meissner A. (2013). DNA methylation: Roles in mammalian development. Nat. Rev. Genet..

[134] Kopp F., Mendell J.T. (2018). Functional Classification and Experimental Dissection of Long Noncoding RNAs. Cell.

[135] Yang Y., Chen L., Gu J., Zhang H., Yuan J., Lian Q., Lv G., Wang S., Wu Y., Yang Y.T. (2017). Recurrently deregulated lncRNAs in hepatocellular carcinoma. Nat. Commun..

[136] Lye S.H., Polycarp N., Durojaye T.J., Tollefsbol T.O. (2025). Functional Heterogeneity and Context-Dependent Roles of LncRNAs in Breast Cancer. Cancers.

[137] Chakravarti R., Lenka S.K., Gautam A., Singh R., Ravichandiran V., Roy S., Ghosh D. (2022). A Review on CRISPR-mediated Epigenome Editing: A Future Directive for Therapeutic Management of Cancer. Curr. Drug Targets.

[138] Ansari I., Chaturvedi A., Chitkara D., Singh S. (2022). CRISPR/Cas mediated epigenome editing for cancer therapy. Semin. Cancer Biol..

[139] Casado-Pelaez M., Bueno-Costa A., Esteller M. (2022). Single cell cancer epigenetics. Trends Cancer.

[140] Song J., Yang P., Chen C., Ding W., Tillement O., Bai H., Zhang S. (2025). Targeting epigenetic regulators as a promising avenue to overcome cancer therapy resistance. Signal Transduct. Target. Ther..

